# Infiltrative Optic Neuropathy in Advanced Breast Carcinoma

**DOI:** 10.7759/cureus.50994

**Published:** 2023-12-23

**Authors:** Nurul Farah H Shahrudin, Julieana Muhammed, Wan-Hazabbah Wan Hitam

**Affiliations:** 1 Ophthalmology, Universiti Sains Malaysia, Kota Bharu, MYS

**Keywords:** radiotherapy, chemotherapy, breast carcinoma, orbital metastases, infiltrative optic neuropathy

## Abstract

Infiltrative optic neuropathy is a condition characterized by the invasion of tumor cells into the optic nerve. Breast carcinoma can metastasize to various organs, most commonly the bones, lungs, and liver, and rarely involves the orbit. Orbital involvement may result in debilitating visual impairment and blindness. We report a case of infiltrative optic neuropathy secondary to advanced breast carcinoma. A 39-year-old woman with stage 4 breast carcinoma presented with sudden-onset blurred vision in her right eye for one week. It was associated with a localized scotoma in the visual field. She was previously diagnosed with secondary metastases involving the liver and bone and is currently undergoing treatment with chemotherapy and radiotherapy. Visual acuity in the right eye was 6/7.5, with a positive relative afferent pupillary defect and an inferonasal field defect. The extraocular muscle movement was full, with no significant proptosis. Both anterior segments were unremarkable. Fundoscopy showed a normal optic disc in both eyes, with no optic disc swelling. A computed tomography (CT) scan of the brain and orbit revealed secondary metastases in the dura and right orbital apex. Magnetic resonance imaging (MRI) of the brain revealed right infiltrative optic neuropathy. The patient received whole-brain radiotherapy (WBRT), followed by 12 cycles of chemotherapy. On follow-up, the patient was stable; however, her vision in the right eye deteriorated from 6/7.5 to perception of light. In conclusion, orbital metastasis should be the leading diagnostic consideration when the affected patient has a history of cancer. Early detection, coupled with prompt treatment, can help patients achieve better visual outcomes and, whenever possible, preserve their vision.

## Introduction

Breast carcinoma is one of the most common cancers worldwide, with a significantly high risk of metastasis. Many cases of ocular and orbital metastases have been documented to have originated from breast cancer [1]. The frequency of ocular involvement is reported to be as high as 30% in patients with known metastatic breast disease. In a case series study, Shields et al. reported that 4.5% of patients with ocular metastases had metastases to the optic nerve [2]. Infiltrative optic neuropathy involves the infiltration of cancer cells from the primary tumor, which can result in structural changes and direct or indirect compression of the optic nerve.

The diagnosis of orbital metastases poses a significant challenge to physicians because the ocular manifestations of breast neoplasia are numerous and non-specific. A cancer patient is at risk of metastasis for the rest of their life. It was reported that nearly 54% of patients with orbital breast metastases present with infiltrative or inflammatory signs [3]. Other possible diagnostic considerations that need to be excluded are ocular toxicities-induced optic neuropathy secondary to the chemotherapeutic agents. The side effects of the treatment are generally not preventable; therefore, clinicians must be aware of the possibility of potential vision-threatening complications [4]. A definitive diagnosis can only be made after a thorough history and physical examination, with particular attention to the patient's history of breast cancer. We report a case of infiltrative optic neuropathy in orbital metastases from breast carcinoma.

## Case presentation

A 39-year-old woman with breast carcinoma presented with blurred vision in her right eye for a one-week duration. It was described as a central blurring of vision and was associated with a headache. She was initially diagnosed with right invasive ductal breast carcinoma seven years ago when she presented with a breast mass. She denied any positive family history of cancer in the family. She is a full-time homemaker and was blessed with three children. Computed tomography of the thorax, abdomen, and pelvis done at that time showed no evidence of metastasis. She underwent a right breast mastectomy with axillary clearance. The tumor biopsy was reported as estrogen receptor (ER)/progesterone receptor (PR) positive and human epidermal growth factor 2-neu (HER 2-neu) positive. She received 15 cycles of radiotherapy and eight cycles of chemotherapy (doxorubicin and cyclophosphamide). During her two-year follow-up, it was discovered that she had developed distant metastases in her liver and bones. She was restarted on a second-line chemotherapy treatment (Zometa and docetaxel).

**Figure 1:**
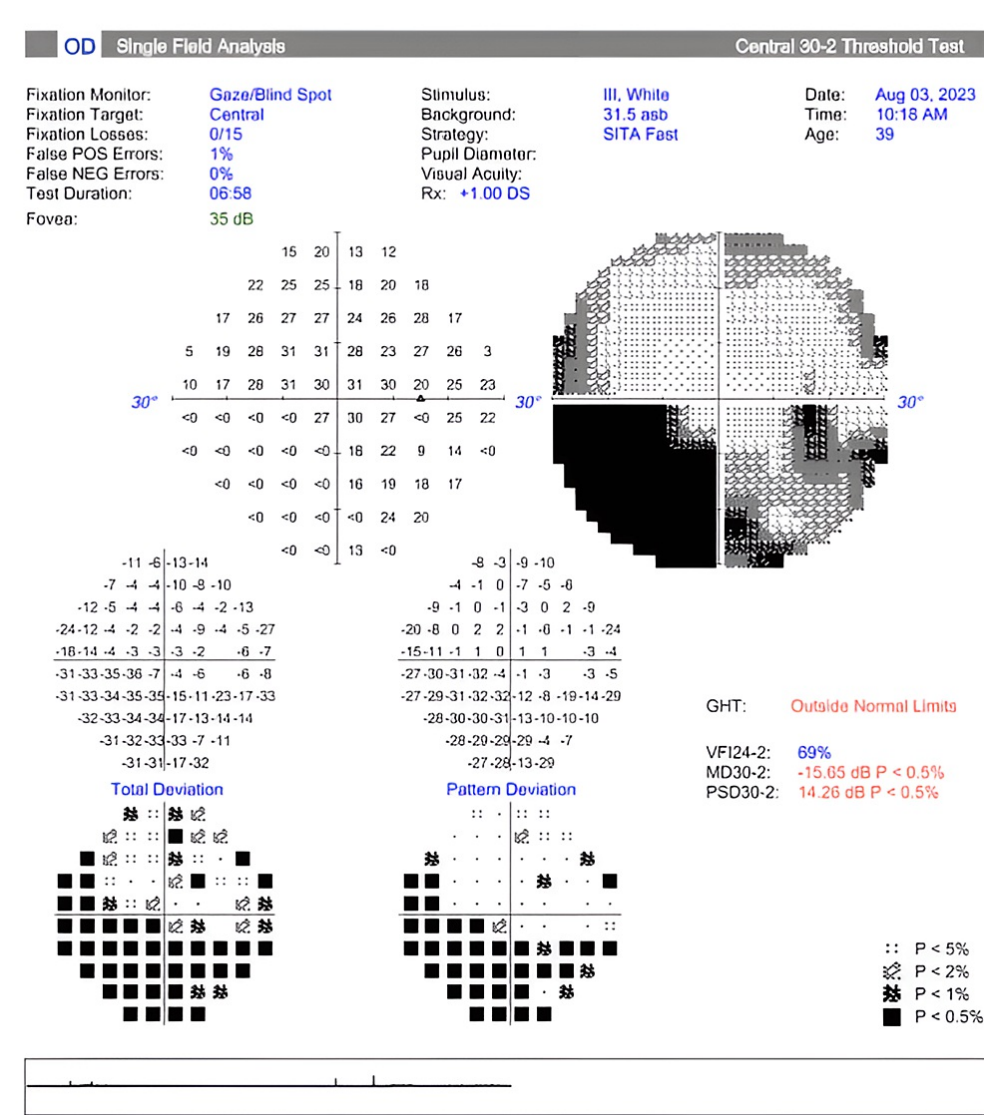
The HVF report of the right eye shows an inferonasal quadrantanopia field defect. HVF: Humphrey visual field

Visual acuity in the right eye was 6/7.5, and in the left eye, it was 6/6. The Relative Afferent Pupillary Defect (RAPD) was positive. There was a significant reduction in the optic nerve function tests of the right eye, in which the red saturation and light brightness were reduced to 80% and 70%, respectively. The color vision was also reduced, scoring 14 out of 15 using the Ishihara chart. The extraocular muscle movement was full, with no significant proptosis. Both anterior segments were unremarkable, and the intraocular pressure was normal with a reading of 10 mmHg for both eyes. Fundoscopy showed a normal appearance of the optic disc and retina, with no optic disc swelling. The Humphrey visual field (HVF) test showed an inferonasal visual field defect (Figure 1).

**Figure 2:**
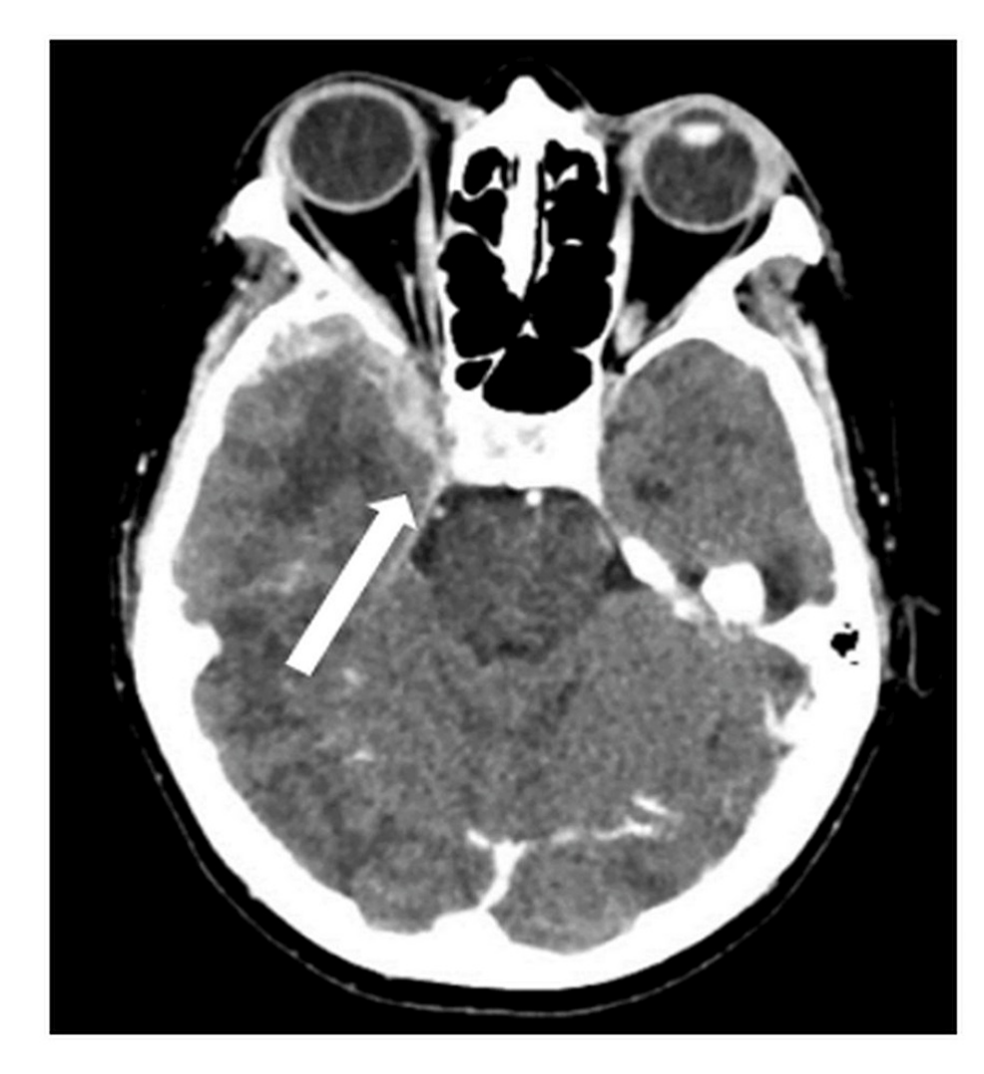
A contrast-enhanced CT scan and orbit (axial view) showed the presence of a lesion seen along the dura of the right frontal and right temporal regions, approaching the right orbital apex.

The optical coherence tomography of the optic nerve head was normal in both eyes. Her workup for the blood investigation showed moderate anemia with a hemoglobin level of 9.6 grams per deciliter; otherwise, other parameters such as white cell count, platelet count, and renal function test were normal. However, her liver function tests were slightly deranged, likely due to the presence of liver metastasis. A contrast-enhanced CT scan of the brain and orbit (Figure 2) showed multiple intracranial lesions involving the dura and right orbital apex, suggestive of metastasis.

**Figure 3:**
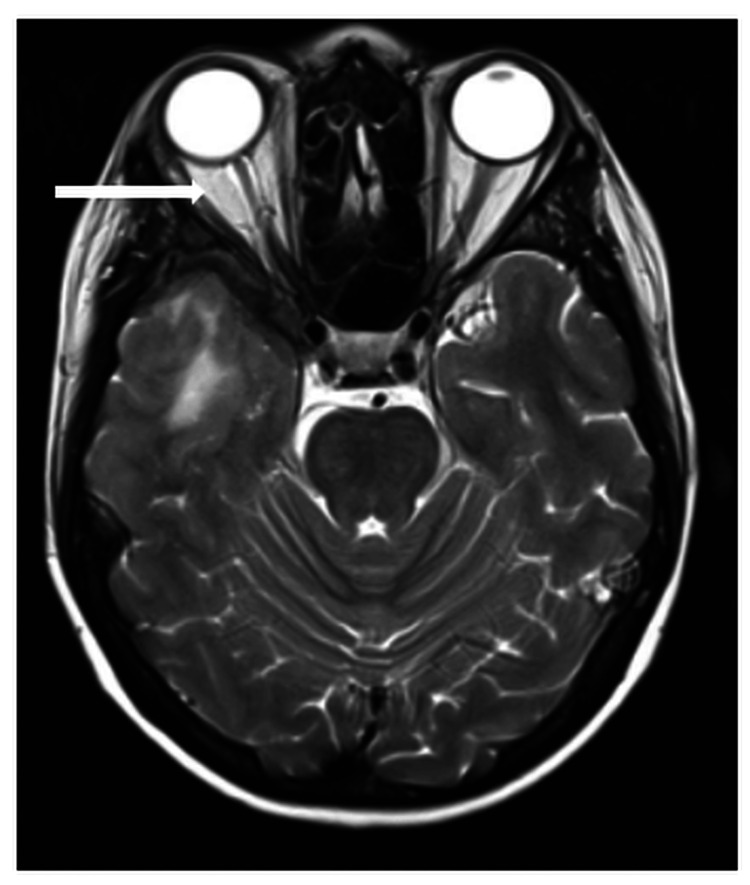
An MRI of the brain (axial view, T2 sequence) showed the presence of enhancement involving the right optic nerve.

Magnetic resonance imaging revealed a right frontal dural lesion with extension to the right optic nerve. The intracanalicular and intracranial parts of the right optic nerve were enhanced (Figure 3).

She was diagnosed with infiltrative optic neuropathy in her right eye, which was secondary to orbital metastases from breast carcinoma.

The patient was co-managed by a multidisciplinary team. The patient was started on a course of whole-brain radiotherapy (WBRT) consisting of five cycles. Then, 12 cycles of the carboplatin and gemcitabine (carbo Gemzar) chemotherapy regimen were administered. There was no active management by the neurosurgery and general surgery teams due to her advanced disease status.

The patient noticed a worsening of right eye vision after completing WBRT. Her visual acuity in her right eye reduced from 6/7.5 to perception of light. Then the patient proceeded with chemotherapy treatment. Her vision improved to the point of counting fingers after receiving six cycles of chemotherapy. The left eye remained stable. Systemically, this patient is generally still ambulating well with no other neurological deficit elsewhere. She still has six cycles of chemotherapy to complete. Apart from being managed primarily by the oncology team, this patient was also under the medical and surgical team for monitoring of her blood parameters and systemic condition, with regular two- to three-monthly follow-ups.

## Discussion

Infiltrative optic neuropathy from breast cancer can cause severe visual impairment. Metastases can reach the optic nerve through various routes, including the choroid, vascular dissemination, invasion from orbit, or via the central nervous system. It is reported that the choroid is the most common site, presumably because of its large blood supply. The involvement of the optic nerve is much less common. The lesion may arise as an extension from choroidal, central nervous system, or orbital metastases, or it may be found as an isolated site. The isolated lesions are extremely rare, occurring in three out of 227 (1.3%) cases [5].

The onset of orbital involvement varies depending on the case, with some cases occurring early in the disease while others occur at a late stage. It was previously reported that the time interval between the onset of breast carcinoma diagnosis and orbital metastasis development had a wide range, spanning from one month to 25 years [6]. The latest study in 2020 reported that orbital involvement occurs on average 4.5 to 6.5 years after primary tumor diagnosis [7]. This is similar to our case, where the patient was diagnosed with orbital involvement six years after the initial diagnosis of breast cancer.

Neuroimaging techniques such as CT scans and MRI are very useful tools used in diagnosing orbital and brain metastases. Because most orbital metastases mainly affect the orbital soft tissues, MRI usually provides the best resolution for detecting orbital metastases [2]. Almost all orbital metastases evaluated with MRI show some degree of enhancement with contrast agents [8]. Enlargement of the lesion is an important MRI finding in neoplastic disorders, including optic nerve infiltration. An MRI of the brain and orbit may show meningeal and optic nerve enhancements [9]. In our case, the MRI showed the presence of a right frontal dural lesion with extension to the right optic nerve. The intracanalicular and intracranial parts of the right optic nerve, as well as the subarachnoid space, are prominent and demonstrate high signal intensity.

The primary goal of treatment should be to remove the cancerous tumor cells and prevent further spread and damage to other organs. However, orbital metastases indicate that the disease is already advanced at the time of diagnosis and is not curable. Therefore, orbital surgery is typically used for diagnostic purposes rather than therapeutic ones [8]. In this case, upon the patient's recent diagnosis of brain metastases, surgical removal of the brain metastases was not considered, given the advanced disease stage. Moreover, there are other treatments offered, such as starting the patient on WBRT to target the brain metastases, followed by radiotherapy. Breast cancer metastases are radiosensitive, and external beam radiotherapy is the most established treatment [10]. Radiotherapy is now a widely available treatment modality. A previous study by Cohen et al. found that this treatment stabilized or restored vision in up to 86% of patients [11]. Previous studies have reported that chemotherapy provides a large reduction in the risk of recurrence for patients with hormone receptor (HR)-positive breast cancer [9]. Early diagnosis and treatment of cancer patients with known metastases can help provide relief and improve their quality of life.

Patients who have been diagnosed with orbital metastases usually have a poor prognosis, with a mean survival of about 31 months (ranging from one to 116 months) after diagnosis [12]. Shields et al. reported in 2001 that the five-year survival rate of patients with metastatic breast cancer is 21% [2].

## Conclusions

Infiltrative optic neuropathy in the setting of orbital metastases from breast carcinoma poses significant challenges for healthcare providers, as it usually indicates advanced disease with a guarded prognosis. Early recognition, accurate diagnosis, and a multidisciplinary approach are essential for optimizing patient outcomes and helping to improve the quality of life. We believe that this case can help to increase awareness and insight among healthcare practitioners who encounter similar scenarios in the future.
